# ReConPlot: an R package for the visualization and interpretation of genomic rearrangements

**DOI:** 10.1093/bioinformatics/btad719

**Published:** 2023-12-07

**Authors:** Jose Espejo Valle-Inclán, Isidro Cortés-Ciriano

**Affiliations:** European Molecular Biology Laboratory, European Bioinformatics Institute, Wellcome Genome Campus, Hinxton, Cambridge CB10 1SD, United Kingdom; European Molecular Biology Laboratory, European Bioinformatics Institute, Wellcome Genome Campus, Hinxton, Cambridge CB10 1SD, United Kingdom

## Abstract

**Motivation:**

Whole-genome sequencing studies of human tumours have revealed that complex forms of structural variation, collectively known as complex genome rearrangements (CGRs), are pervasive across diverse cancer types. Detection, classification, and mechanistic interpretation of CGRs requires the visualization of complex patterns of somatic copy number aberrations (SCNAs) and structural variants (SVs). However, there is a lack of tools specifically designed to facilitate the visualization and study of CGRs.

**Results:**

We present **ReConPlot** (**RE**arrangement and **CO**py **N**umber **PLOT**), an R package that provides functionalities for the joint visualization of SCNAs and SVs across one or multiple chromosomes. ReConPlot is based on the popular ggplot2 package, thus allowing customization of plots and the generation of publication-quality figures with minimal effort. Overall, ReConPlot facilitates the exploration, interpretation, and reporting of CGR patterns.

**Availability and implementation:**

The R package ReConPlot is available at https://github.com/cortes-ciriano-lab/ReConPlot. Detailed documentation and a tutorial with examples are provided with the package.

## 1 Introduction

The advent of whole genome sequencing (WGS) has enabled a more nuanced characterization of the diversity, rates and underlying mechanisms of chromosomal alterations than was ever possible using cytogenetic or pathology analyses (Greenman *et al.* 2007, Mardis and Wilson 2009, Garraway and Lander 2013, Cortés-Ciriano *et al.* 2021). WGS studies of human cancers have revealed that genomic instability, a hallmark of cancer, manifests as alterations in the structure and number of chromosomes (aneuploidy), whole genome doubling, repeat instability, and remarkably diverse forms of structural variants (SVs) (Macintyre *et al.* 2018, Priestley *et al.* 2019, ICGC/TCGA Pan-Cancer Analysis of Whole Genomes Consortium 2020, Steele *et al.* 2022). SVs, which account for most driver events in some cancer types (Zack *et al.* 2013, ICGC/TCGA Pan-Cancer Analysis of Whole Genomes Consortium 2020), refer to the rearrangement of the genome leading to the deletion, amplification or reshuffling of genomic segments. In cancer genomes, genomic rearrangements manifest as (i) simple events, such as deletions, duplications, inversions, and insertions occurring in isolation, or (ii) complex events involving multiple breakpoints across one or multiple chromosomes and showing complex patterns of both spatial and temporal clustering (Cortés-Ciriano *et al.* 2020, Li *et al.* 2020, Hadi *et al.* 2020, Bao *et al.* 2022). Such complex patterns, collectively referred to as complex genomic rearrangements (CGRs), include those recently discovered in cancer genome studies, such as chromothripsis (Stephens *et al.* 2011, Rausch *et al.* 2012, Cortés-Ciriano *et al.* 2020), chromoanasynthesis (Liu *et al.* 2011), chromoplexy (Baca *et al.* 2013), pyrgo, rigma, and tyfonas (Hadi *et al.* 2020), as well as others initially described in cytogenetic studies, such as breakage–fusion–bridge cycles (Campbell *et al.* 2010) and double minutes or extrachromosomal DNA elements (Turner *et al.* 2017, Deshpande *et al.* 2019). Multiple algorithms have been developed to detect and classify CGRs (Notta *et al.* 2016, Cortés-Ciriano *et al.* 2020, Hadi *et al.* 2020, Bao *et al.* 2022) based on the analysis of the patterns of SVs and somatic copy number aberrations (SCNAs) detected through computational cancer genome analysis. However, due to the diversity, complexity, variable scale and overlapping features of CGRs, coupled to their co-localization (Cortés-Ciriano *et al.* 2020), their detection and classification remains a challenging task. As a result, manual inspection of SCNA and SV data is often required to resolve the most complex cases (Li *et al.* 2014, Notta *et al.* 2016, Cortés-Ciriano *et al.* 2020). This task requires versatile methods to visualize SCNAs and SVs across genomic regions ranging from a few kbp to multiple chromosomes. A popular approach for genomics data visualization, termed Circos plot (Krzywinski *et al.* 2009), allows exploration of CGRs by displaying the cancer genome in a circular layout where concentric circles show different types of mutations and genomic features (Nusrat *et al.* 2019). Although versatile to provide an overview of the cancer genome (Davies *et al.* 2017, Goldman *et al.* 2020, Shale *et al.* 2022), Circos plots are often too complex to visualize CGRs involving large numbers of SVs and SCNAs. An alternative approach consists of displaying genomic regions of interest in a linear layout where regions of equal copy number are represented by segments, and SVs by arcs (Li *et al.* 2014, ICGC/TCGA Pan-Cancer Analysis of Whole Genomes Consortium 2020). This visualization strategy, usually referred to as genomic rearrangement plots, has become popular in the cancer genomics community for visualizing and reporting the patterns and consequences of CGRs (e.g., disruption of tumour suppressor genes by SVs) (Cortés-Ciriano *et al.* 2020, ICGC/TCGA Pan-Cancer Analysis of Whole Genomes Consortium 2020). However, there is lack of easy-to-use software packages to visualize genomic rearrangement profiles and generate publication-quality figures for reporting cancer genome analysis results. Here we present **ReConPlot** (**RE**arrangement and **CO**py **N**umber **PLOT**), an R package that provides functionalities for the joint visualization of SCNAs and SVs across one or multiple chromosomes.

## 2 Methods

**Figure 1.**
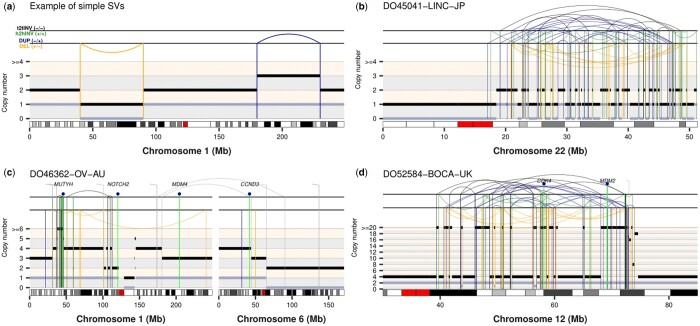
Examples of ReConPlots visualizing complex genomic rearrangements detected in four cancer genomes from the PCAWG cohort. (a) Artificial example of a simple deletion (left, orange) and a simple duplication (right, blue). (b) Example of a canonical chromothripsis event detected in a liver adenocarcinoma. The ReConPlot shows the characteristic cluster of interleaved SVs and copy number oscillations between two copy number states accompanied by loss of heterozygosity, which is indicated by the regions with a minor copy number of 0. (c) Example of a CGR spanning two chromosomes detected in an ovarian adenocarcinoma. (d) Example of a CGR showing high-level amplifications detected in an osteosarcoma genome. The chromothripsis event occurred after whole-genome doubling, as evidenced by the minor copy number oscillations between copy number states 1 and 2, and caused the high-level amplification of *CDK4* and *MDM2*. Tail-to-tail (t2tINV) inversions, head-to-head (h2hINV) inversions, duplication-like SVs (DUP), and deletion-like SVs (DEL) are depicted in black, green, blue, and orange, respectively. Total and minor copy number values are represented by black and grey segments, respectively. ICGC IDs are shown on top of each ReConPlot.

ReConPlot relies on the popular package ggplot2 (Wickham 2016) for the visualization of SCNA and SV profiles, thus allowing for user-specific customization and integration with functionalities from other R packages to compose multi-panel figures easily. The main function of the package, *ReConPlot*, only requires as input the genomic coordinates for the regions to be visualized, integer minor and total copy number data, and SV information in browser extensible data paired-end (BEDPE) format (Quinlan and Hall 2010). *ReConPlot* permits the visualization of genomic rearrangement profiles across one or multiple chromosomes (Fig. 1).

Each ReConPlot consists of three main panels. The bottom panel shows Giemsa binding data (Cheung *et al.* 2001, Furey and Haussler 2003) for the genomic regions of interest. The middle panel reports total and minor copy number information. Finally, the top panel shows SVs. SVs whose breakpoints fall within the regions selected to be displayed are represented by arcs. In cases where only one breakpoint maps to the selected genomic regions, the SV is represented as a vertical line ending with a 45-degree overhang. SVs are categorized into four groups depending on the read orientation at the breakpoints (i.e. type of fragment joins) following the notation established by the Pan-Cancer Analysis of Whole Genomes project [PCAWG (ICGC/TCGA Pan-Cancer Analysis of Whole Genomes Consortium 2020); Fig. 1]: deletion-like SVs (DEL) are represented as ‘+ −’, duplication-like SVs (DUP) as ‘− +’, tail-to-tail inversions (t2tInv) as ‘− −’, and head-to-head inversions (h2hInv) as ‘+ +’. Using the same notation as PCAWG allows for smooth integration with other software packages designed for the detection, classification and interpretation of CGR, such as *ShatterSeek* (Cortés-Ciriano *et al.* 2020). In addition, *ReConPlot* provides functionalities to highlight the location of genes (see Fig. 1 for examples). Currently, ReConPlot supports the following builds of the human reference genome: GRCh37, GRCh38, and T2T-CHM13, and the mouse reference genome: mm10, mm39. While default parameter values ensure the generation of publication-quality figures, the function *ReConPlot* is highly versatile, as it allows customization of the layout of the plots, font sizes, font colours, and other graphical parameters (see the documentation and tutorial of the package for a full list of customizable graphical options). Additionally, an optional module is available to depict a user-defined annotation below the chromosome ideogram with aligned genomic coordinates. The optional annotation module can be used to, for example, annotate point mutations of interest and their variant allele frequency, or any other genomic features of interest to the user.

## 3 Results

We have extensively validated the functionalities of ReConPlot using SV and SCNA calls from the PCAWG project, allowing us to identify and classify diverse CGRs, such as chromothripsis, CGRs involving multiple chromosomes, and CGRs showing high-level oncogene amplifications (Fig. 1). In sum, ReConPlot provides functionalities for the visualization and interpretation of complex genomic rearrangement profiles detected in cancer genomes and rare disease patients.
